# Perception of cancer in patients diagnosed with the most common gastrointestinal cancers

**DOI:** 10.1186/s12904-020-00650-w

**Published:** 2020-09-17

**Authors:** Aleksandra Czerw, Urszula Religioni, Tomasz Banaś

**Affiliations:** 1 Department of Health Economics and Medical Law, Medical University of Warsaw, Warsaw, Poland; 2 Department of Economic and System Analyses, National Institute of Public Health – NIH, Warsaw, Poland; 3 Collegium of Business Administration, Warsaw School of Economics, Warsaw, Poland; 4 Department of Gynaecology and Oncology, Jagiellonian University Medical College, Krakow, Poland

**Keywords:** Gastrointestinal cancer, Illness acceptance, Quality of life, Strategies for coping with the illness

## Abstract

**Background:**

Gastrointestinal cancers, including colorectal cancer, stomach cancer and pancreatic cancer are among the most common cancers in Poland. Cancer patients usually assess their quality of life much worse than the general population, while negative emotions associated with the illness may affect the results of treatment.

**Methods:**

The study involved 378 patients with colorectal cancer, stomach cancer and pancreatic cancer, treated as outpatients at the Oncology Center - Maria Skłodowska-Curie Institute in Warsaw in 2013–2018. Standardized tools were used in the study: the Beliefs about Pain Control Questionnaire (BPCQ), the Pain Coping Strategies Questionnaire (CSQ), Approval Illness Scale (AIS), Mental Adjustment to Cancer (MiniMAC). The main goal of the study was to assess pain control, pain management strategies, illness acceptance and adaptation to cancer in patients with the most common gastrointestinal cancers.

**Results:**

Patients with gastrointestinal cancers ascribe the greatest role in controlling pain to internal factors (M = 16.84, SE = .34), and the highest score in this area was obtained by patients with colorectal cancer (M = 17.33, SE = .35). The most frequently chosen strategy is declaring coping (M = 20.95, SE = .57), although patients with pancreatic cancer obtained a high score also in the area of catastrophizing (M = 17.99, SE = 1.14). The average value of illness acceptance for patients with gastrointestinal cancers was M = 25.00 (SE = .50) and it was the lowest in the group of patients diagnosed with pancreatic cancer (M = 23.41, SE = 1.16), and the highest in a group of people with colorectal cancer (M = 27.76, SE = .51). Patients with gastrointestinal cancers obtained the highest values of the MiniMAC test in the area of the fighting spirit (M = 21.30, SE = .25), characteristic mainly for patients with colorectal cancer. Patients with pancreatic cancer were characterized by high anxiety and helplessness/hopelessness.

**Conclusions:**

Patients with gastrointestinal cancers use different methods of pain control and pain coping strategies, with active behaviors being preferred by patients with colorectal cancer and destructive - by patients with pancreatic cancer. The majority of socio-economic variables, as well as the treatment method, affect the patients’ behaviors.

## Background

Cancer, especially at its advanced stage, affects both the reduction of the physical functioning of the patient (mainly due to the strong pain experienced), but it also affects psychological aspects. Cancer patients usually assess their quality of life much worse than the general population, and this assessment may be related to both the severity of the illness and various sociodemographic features [1].

Gastrointestinal cancers are among the most common in Poland. Colorectal cancer is the second most common cancer in women (after breast cancer) and the third in men (after lung and prostate cancer). In terms of mortality, it is the second most common cause of cancer deaths among men (after lung cancer) and the third most common among women (after lung and breast cancer). Generally, the standardized incidence rate of colorectal cancer in Poland is 23.9/100,000 people, and mortality rate 14.0/100,000 people [2].

Stomach cancer is the fifth most common cancer in men, being the fourth most common cause of death due to cancer among this group of Poles. Currently, the standardized rate for developing stomach cancer in Poland is 7.7/100,000 people, and the mortality rate is 7.4/100,000 people [3].

Pancreatic cancer is the fifth leading cause of death in women and the sixth in men due to cancer. The standardized incidence rate for pancreatic cancer in Poland is 4.8/100,000 people, and the mortality rate - 6.4/100,000 people [4].

Limitations resulting from the treatment of gastrointestinal cancer can significantly affect the quality of life of patients. The acceptance of the diagnosis of cancer and related discomfort of life, pain, and reduced independence is of crucial importance in this respect. Cancer affects many aspects of functioning, both the physical and mental spheres as well as the functioning in society.

In turn, stress or anxiety that patients may feel at any stage of diagnosis or treatment may affect each of these spheres, causing the intensification of many negative emotions associated with the illness in patients. However, studies indicate that a passive or destructive approach to the illness significantly reduces quality of life assessed by patients, and many sources confirm that the approach to cancer significantly affects the results of treatment [5, 6].

The main objective of the study was to assess pain control, pain coping strategies, acceptance of illness and psychological adaptation to cancer in patients with the most common gastrointestinal cancers in Poland. The influence of socioeconomic variables on the obtained results as well as the dependence of these results on the diagnosis of metastases and the type of treatment applied were also examined.

## Methods

The study was conducted between 2013 and 2018 among representative sample of 378 patients with gastrointestinal cancers being outpatients of the Oncology Center – Maria Skłodowska-Curie Institute in Warsaw. Inclusion criteria involve:

- age over 18 years,
- diagnosis of gastrointestinal cancer (stomach, colorectal or pancreatic cancer),
- ongoing cancer treatment (chemotherapy, radiotherapy, targeted therapy).

All patients approached the study met the inclusion criteria and were included in the study.

The exclusive criterion was the diagnosis of another cancer in the patient.

### Measures

The following four psychometric tests were used in the study:

1. The Beliefs about Pain Control Questionnaire (BPCQ), designed to test people suffering from pain [7] - the questionnaire consists of 13 statements drawn from three dimensions, which measure the strength of individual beliefs about pain control: personally (internal), the influence of doctors (others) or random events. Each statement is available on a response scale of 1–6, where one means “no, I totally disagree”, six – “yes, I totally agree”. For each dimension of the BPCQ study, the sum of the results is calculated separately based on the sum of points obtained in the selected instructions. The higher the score, the greater the approximation effect on the patient’s pain control.
2. The Pain Coping Strategies Questionnaire (CSQ), used to examine people who complain of pain [8] - the questionnaire consists of 42 statements, assessed on a scale of 0–6. The points comprise six cognitive strategies (diverting attention, reinterpreting pain sensations, catastrophizing, ignoring pain, praying/hoping, coping self-statements) and one behavioral strategy (increasing behavioral activity). The score for each strategy ranged from 0 to 36 points. The higher the score, the more significant the factor in the process of coping with pain.
3. Acceptance Illness Scale (AIS), measures the level of adaptation to the illness [9] - the scale consists of eight statements on the adverse effects of poor health (restrictions imposed by the disease, a feeling of dependence on others, decreased self-esteem and lack of self-sufficiency). The eight statements on the use of AIS are each assigned a scale from 1 to 5, where one stands for “I strongly agree” and five – “I strongly disagree”. The patient can score between 8 and 40 points, which takes into consideration the degree of disease acceptance.
4. Mental Adaptation to Cancer (Mini-MAC), measuring the level of mental adjustment to cancer [10] - the questionnaire consists of 29 statements highlighting four ways of coping with the disease: anxious preoccupation, fighting spirit, helplessness-hopelessness, and positive reassessment. While anxiety and helplessness-hopelessness shape a passive (destructive) style of dealing with the disease, fighting spirit and positive reassessment impact on the active (constructive) type of dealing with the illness. For every Mini-MAC statement, there are four response scales from 1 (definitely no) to 4 (definitely yes). Points in each strategy are computed separately subject to a series of numbers derived in the analysis results, and for which the final score may range from 7 to 28 points. The higher the score, the greater the coping strategy.

Patients were also asked about socio-economic data: gender (woman/man), education (basic, vocational, secondary, higher), place of residence (village, a city of up to 20,000 inhabitants, up to 50,000, up to 100,000, up to 500,000 and a city with over 500,000 inhabitants), average monthly income (below PLN 500, PLN 501–1000, PLN 1001-1500, PLN 1501-2000, over PLN 2000), professional status (working person, student, retired, housekeeper, unemployed), marital status (single, married, widow/widower, divorced / divorcee), as well as the presence of metastasis (yes/no) and the type of treatment used (chemotherapy, radiotherapy, targeted therapy).

The study used the PAPI technique (Paper and Pencil Interview).

The obtained results were subjected to statistical analysis using t Student for independent samples, U Mann-Whitney test, H Kruskall-Wallis test, one-way analysis of variance and Pearson and Spearman r correlation (in case of age variable). The assumed level of statistical significance is *p* < 0.05. Statistical analysis was performed in two steps. Firstly the associations between socio-medical variables and analysed variables were assessed. The statistically significant associations between socio-medical data and analysed variables were used in the subsequent analysis. The socio-medical variables significantly related to analysed variables were used as covariates in computing adjusted estimated marginal means which were then used in the comparisons between the groups of patients diagnosed with three types of cancer.

## Results

The study involved 378 patients aged 23–91 years (M = 64.26, SD = 12.20), including 168 (44.4%) women aged 23–90 (M = 62.16, SD = 12.27) and 210 (55.6%) men aged 28–91 (M = 65.95, SD = 11.91). Among the studied group of patients, 239 (63.2%) suffered from colorectal cancer, 93 (24.6%) from stomach cancer, and 46 (12.2%) from pancreatic cancer.

Detailed descriptive results regarding the studied socioeconomic and clinical variables in the three groups are presented in Table 1. The table also presents the values of likelihood ratio which was used to verify statistical significance of the associations between the group membership and other variables.

**Table 1:** Characteristics of patients

	Total		Pancreas		Stomach		Large intestine				
Feature	*n*	%	*n*	%	*n*	%	*n*	%	λ	*df*	*p*
**Gender**											
woman	168	44.4	24	52.2	45	48.4	99	41.4	2.58	2	.276
man	210	55.6	22	47.8	48	51.6	140	58.6			
**Education**											
basic	42	11.1	4	8.7	11	11.8	27	11.3	5.33	8	.722
vocational	99	26.2	12	26.1	22	23.7	65	27.2			
secondary	140	37.0	14	30.4	33	35.5	93	38.9			
higher	96	25.4	16	34.8	27	29.0	53	22.2			
No data	1	0.03	0	.0	0	.0	1	.4			
**Place of residence**											
village	69	18.3	5	10.9	19	20.4	45	18.8	21.43	10	.018
city of up to 20,000 inhabitants	48	12.7	8	17.4	9	9.7	31	13.0			
city of up to 50,000 inhabitants	44	11.6	3	6.5	5	5.4	36	15.1			
city of up to 100,000 inhabitants	47	12.4	10	21.7	10	10.8	27	11.3			
city up to 500,000 inhabitants	36	9.5	8	17.4	12	12.9	16	6.7			
city over 500,000 inhabitants	134	35.4	12	26.1	38	40.9	84	35.1			
**Average monthly income**											
no data	4	1.1	0	.0	0	.0	4	1.7	11.01	12	.528
below PLN 500	7	1.9	1	2.2	3	3.2	3	1.3			
PLN 501–1000	85	22.5	8	17.4	23	24.7	54	22.6			
PLN 1001–1500	114	30.2	14	30.4	22	23.7	78	32.6			
PLN 1501–2000	87	23.0	12	26.1	19	20.4	56	23.4			
over PLN 2000	81	21.4	11	23.9	26	28.0	44	18.4			
**Professional status**											
working person	135	35.7	20	43.5	36	38.7	79	33.1	6.68	8	.571
student	5	1.3	0	.0	3	3.2	2	.8			
pensioner	213	56.3	23	50.0	49	52.7	141	59.0			
housekeeper	18	4.8	2	4.3	3	3.2	13	5.4			
unemployed	7	1.9	1	2.2	2	2.2	4	1.7			
**Marital status**											
single	15	4.0	3	6.5	4	4.3	8	3.3	3.95	6	.684
married	271	71.7	29	63.0	65	69.9	177	74.1			
widow / widower	65	17.2	9	19.6	19	20.4	37	15.5			
divorced / divorcee	27	7.1	5	10.9	5	5.4	17	7.1			
**The presence of metastasis**											
Yes	139	36.8	23	50.0	29	31.2	87	36.4	4.63	2	.099
No	239	63.2	23	50.0	64	68.8	152	63.6			
**Type of treatment**											
chemotherapy	98	52.4	25	54.3	45	48.4	128	53.6	.80	2	.671
radiotherapy	74	19.6	15	32.6	21	22.6	38	15.9	7.02	2	.030
targeted treatment	29	7.7	10	21.7	14	15.1	5	2.1	29.11	2	.001

Table 1. Descriptive results of the studied socioeconomic and clinical variables.

There were statistically significant associations between type of cancer and place of residence, being treated with radiotherapy and being treated with targeted treatment. There were more patients living in the cities with over 500,000 inhabitants in the group of patients diagnosed with stomach cancer and in the group of patients diagnosed with large intestine cancer. More patients from the group of patients diagnosed with pancreas were treated with radiotherapy and with targeted treatment.

### Pain control

Analyzing the results of the BPCQ, in all patients with gastrointestinal cancers, the influence of some of the studied socio-economic variables on particular areas of pain control can be noticed. Age statistically significantly affects the average patient results obtained in the areas of the influence of doctors (r = 0.132) and random events (r = 0.242). The obtained positive correlations indicate that the older the patient the higher the average score obtained in both these areas.

Education significantly influences locating pain control in random events (*p* = 0.001). People with primary/vocational education were characterized by the highest scores in this area (M = 16.49, SD = 4.14), and with the increase in education, the average values of this area were decreasing (for patients with secondary education M = 15.4; SD = 4.31, and for patients with higher education M = 14.17, SD = 4.77).

Income also influenced the results obtained by patients in the BPCQ. Patients with lower incomes (up to PLN 1500 net per household member) obtained higher averages in the area of the influence of doctors (*p* = 0.014) and random events (*p* = 0.001) than people with higher incomes. In the case of the influence of doctors, the average result of people with lower incomes was M = 16.90 (SD = 4.60), and with higher income - M = 15.63 (SD = 4.65). In the area of random events, the average for patients with lower income was M = 16.26 (SD = 4.15), and for people with higher income M = 14.64 (SD = 4.64).

Similarly, the results of tests in the areas of the influence of doctors (*p* = 0.003) and random events (*p* = 0.001) were affected by the professional status. The severity of both these areas was higher in the group of non-working people. Working people obtained the average of M = 15.44 (SD = 4.31) in the influence of doctors and the average of M = 14.25 (SD = 4.29) in the area of random events, while in the case of pensioners these values were respectively: M = 16.92 (SD = 4.63) and M = 16.46 (SD = 4.22).

Gender, place of residence, diagnosis of metastases and the fact that the patient undergoes chemotherapy, radiotherapy or targeted treatment during the last year did not affect the results of patients in the BPCQ (in all cases *p* > 0.05).

Patients with gastrointestinal cancers assign the greatest role in controlling pain to internal factors (M = 16.84, SE = .34), and the smallest to random events (M = 15.45, SE = .28). Taking into account the location of the primary lesion, it can be noted that patients with colorectal cancer (M = 17.33, SE = .35) obtain a higher value in the impact of internal factors on pain control than patients with stomach cancer (M = 16.34; SE = .56) or pancreatic cancer (M = 16.85, SD = .79). Similarly, Patients with colorectal cancer (M = 15.55, SE = .28) than and patients with stomach cancer (M = 15.63, SE = .46) get a higher value of random events than or pancreatic cancer (M = 15.18, SE = .64). In the area of the influence of doctors, the differences among patients considering the location of the primary lesion are much smaller. However, due to the significant inequality of the compared groups, the statistical significance of these differences was not calculated (Table 2).

**Table 2:** Results of the BPCQ for patients with gastrointestinal cancers

BPCQ areas	Total		Pancreas		Stomach		Large intestine		η^**2**^
	M	SE	M	SE	M	SE	M	SE	
Internal factors	16.84	.34	16.85	.79	16.34	.56	17.33	.35	0.006
Influence of doctors	16.41	.30	16.78	.68	16.27	.49	16.17	.30	0.002
Random events	15.45	.28	15.18	.64	15.63	.46	15.55	.28	0.001

*M* Estimated marginal mean, *SE* Standard error, η^2^ Absolute impact measure value

### Strategies for coping with pain

Strategies of coping with pain by patients with gastrointestinal cancers were differentiated by gender, age, education, income, professional status, metastasis diagnosis and the fact of chemotherapy, radiotherapy or targeted treatment. Only the place of residence did not influence the pain management strategy adopted by patients (*p* > 0.05).

The patients’ gender conditioned the result obtained in praying/hoping (*p* = 0.002), in which women obtained significantly higher values than men (respectively M = 22.68, SD = 9.11 for women and M = 19.65; SD = 9.47 for men).

The age of patients differentiated the results obtained in the area of catastrophizing (ρ Spearman = − 0.127), where the higher average was obtained by younger patients.

The patients’ education influenced the average values obtained in the areas of distraction (*p* = 0.025), reevaluation of pain sensations (*p* = 0.038) and catastrophizing (0.002). In terms of distractions, the average value in the group of people with primary or vocational education (M = 21.33, SD = 7.80) was higher than the average values obtained in the group of people with secondary education (M = 18.95, SD = 9.10) and in the group of people with higher education (M = 18.68, SD = 7.88). Similarly, the average value of reevaluation of pain sensations was higher in the group of people with primary or vocational education (M = 14.16, SD = 8.57) than in the group with people with secondary education (M = 11.71) or higher (M = 13 26, SD = 8.54) education. Patients with primary/vocational education achieved higher scores in the area of praying/hoping (M = 22.61, SD = 9.19) than patients with secondary education (M = 21.19, SD = 9.50) and higher (M = 18.25, SD = 9.12) education.

Analyzing income, statistically significant differences were observed in the area of distraction (*p* = 0.002), catastrophizing (*p* = 0.023) and praying/hoping (*p* = 0.001). The average intensities of these three strategies were higher in the group of people with income up to PLN 1500 net per person in the household. The average value obtained by this group of patients in the area of distraction was M = 21.03 (SD = 7.93), in the area of catastrophizing M = 13.20 (SD = 8.26), and in the area of praying/hoping M = 23.08 (SD = 8.99). Correspondingly, for people with income above PLN 1500, these values were M = 18.30 (SD = 8.70) for distraction, M = 11.20 (SD = 8.24) for catastrophizing and M = 18.50 (SD = 9.35) for praying/hoping.

The professional status of the patients influenced the result obtained in the area of praying/hoping (*p* = 0.003). The average value obtained in the working group (M = 19.02, SD = 8.69) was lower than the average value obtained in the group of pensioners (M = 22.08, SD = 9.66).

The fact that metastasis was diagnosed in patients significantly affected the average score achieved in the area of catastrophizing (*p* = 0.001). Patients who had metastases diagnosed had a higher average value (M = 14.09, SD = 8.42) than non-metastatic patients (M = 10.96, SD = 8.06).

Similarly, the average value of catastrophizing was statistically significantly higher in the group of people who were undergoing chemotherapeutic treatment (M = 13.37, SD = 8.21) than in the group of people who were not in the course of chemotherapy treatment (M = 11, 10, SD = 8.26) (*p* = 0.009).

Radiotherapy treatment affected the area of reevaluation of pain sensations (*p* = 0.041) and ignoring pain sensations (*p* = 0.045). In both cases, patients undergoing radiotherapy obtained higher average results for these areas (M = 14.35, SD = 7.35) than in the area of reevaluation of pain sensations, and M = 18.31, SD = 8.06 in the area of ignoring pain sensations in relation to patients not subjected to radiotherapy, where the average values were M = 12.67, SD = 9.16 and M = 15.96, SD = 9.24).

Patients undergoing targeted treatment obtained higher average values in the reevaluation of pain sensations (*p* = 0.018), and catastrophizing (*p* = 0.001), which amounted to M = 16.48, respectively; SD = 7.50 and M = 16.72; SD = 6.98. For patients not undergoing targeted therapy, these values were M = 12.69; SD = 8.91 and M = 11.90; SD = 8.31.

Patients with gastrointestinal cancers obtained the highest average in the CSQ in the areas of declaring coping (M = 20.92, SE = .57), praying/hoping (M = 21.65, SE = .58) and increased behavioral activity (M = 20.44, SD = .56). The largest differences between the different groups of patients analyzed in terms of the primary lesion location were observed in the area of catastrophizing, where patients with pancreatic cancer obtained the highest average score (M = 17.99, SE = 1.14), for patients with stomach cancer the average value of catastrophizing was M = 15.02; (SE = .80), and for patients with colorectal cancer it was the lowest - M = 10.13 (SE = .50) (Table 3). Due to the lack of an equal number of patients with pancreatic cancer, stomach cancer and colorectal cancer, statistical tests were not possible to carry out in this respect.

**Table 3:** Results of the CSQ for patients with gastrointestinal cancers

CSQ areas	Total		Pancreas		Stomach		Large intestine		η^**2**^
	M	SE	M	SE	M	SE	M	SE	
Distraction	19.74	.53	19.45	1.23	20.01	.87	19.77	.54	0.001
Revaluation of pain sensations	12.76	.57	12.63	1.33	12.32	.92	13.33	.58	0.002
Catastrophizing	14.38	.49	17.99	1.14	15.02	.80	10.13	.50	0.121
Ignoring sensations	15.75	.58	14.59	1.34	15.57	.93	17.10	.58	0.011
Praying/hoping	21.65	.58	22.46	1.34	22.26	.94	20.22	.59	0.012
Declaring hoping	20.92	.57	19.83	1.32	21.08	.93	21.86	.58	0.006
Increased behavioral activity	20.44	.56	19.67	1.29	20.22	.91	21.42	.57	0.006

*M* Estimated marginal mean, *SE* Standard error; *η*^*2*^ Absolute impact measure value

### Acceptance of illness

The average value of acceptance of illness in the group of women was 25.18 (SD = 8.25) and it was significantly lower than the average value obtained in the group of men M = 27.12 (SD = 8.32) (*p* < 0.05).

The acceptance of illness did not correlate with the age of patients (r (376) = 0.04, *p* > 0.05).

The average value of acceptance of illness in the group of people with primary or vocational education was M = 25.34 (SD = 8.54), in the group of people with secondary education M = 26.81 (SD = 8.13), and in the group of people with higher education it was M = 26.82 (SD = 8.33), however these differences were not significant (*p* > 0.05).

The average value of acceptance of illness in the group of people living in towns with a population of up to 100,000 was M = 25.69 (SD = 8.26) and it was close to the average value obtained in the group of people who lived in cities with a population of over 100,000 amounting to M = 26.95 (SD = 8.40), which also constitutes a statistically insignificant difference (p > 0.05).

The average value of illness acceptance in the group of people with net income of up to PLN 1500 was M = 25.31 (SD = 8.00) and it was lower than in the group of people who achieved income over PLN 1500.00 M = 27.40 (SD = 8, 60) (*p* < 0.05).

The average value of illness acceptance in the working group was M = 27.54 (SD = 8.15) and it was statistically significantly higher than the average value obtained in the group of pensioners amounting to M = 25.73 (SD = 8.44) (p < 0.05).

In the group of people diagnosed with metastases the average value of illness acceptance was M = 24.26 (SD = 7.43) and it was significantly lower than the average value obtained in the group of people who did not have metastases of M = 27.48 (SD = 8.60) (*p* < 0.001).

The average value of acceptance of illness in the group of people who were undergoing chemotherapy treatment was M = 25.56 (SD = 7.76) and it was close to the average value obtained in the group of people who were not undergoing chemotherapy treatment at M = 27.03 (SD = 8.88), which was a non-statistically significant result (*p* > 0.05). Similarly, average values of acceptance of illness in patients undergoing and not undergoing radiotherapy were similar and were respectively M = 26.23 (SD = 7.40) and M = 26.27 (SD = 8.56) (*p* > 0.05). Also, those who were undergoing targeted treatment obtained a similar average result in the AIS test (M = 24.31, SD = 5.76) as those who were not subjected to targeted therapy (M = 26.41, SD = 8.51) (p > 0.05).

The average value of illness acceptance for patients with gastrointestinal cancers was M = 25.00 (SE = .50) and was lower in the group of patients diagnosed with pancreatic cancer (M = 23.41, SE =1.16) and in the group of patients diagnosed with stomach cancer (M = 23.82, SE = .81), than in a group of people with colorectal cancer (M = 27.76, SE = .51) (Table 4).

**Table 4:** Results of the AIS test for patients with gastrointestinal cancers

AIS area	Total		Pancreas		Stomach		Large intestine		η^**2**^
	M	SE	M	SE	M	SE	M	SE	
Acceptance of illness	25.00	.50	23.41	1.16	23.82	.81	27.76	.51	0.06

*M* Estimated marginal mean, *SE* Standard error, η^2^ Absolute impact measure value

### Mental adaptation to the illness

Among the variables studied, the patients’ gender, age, education, professional status, diagnosis of metastasis, chemotherapeutic treatment and targeted treatment influence mental adaptation to cancer. Place of residence, income, and treatment with radiotherapy did not affect the mental adjustment of patients to the illness (*p* > 0.05).

Women obtained a higher score (M = 22.05, SD = 2.99) in the area of positive reevaluation in comparison to men (M = 21.31, SD = 3.32) (*p* = 0.048).

The age of patients positively correlated with the average values obtained in the area of fighting spirit (ρ Spearman = 0.101) and positive reevaluation ((ρ Spearman = 0.188).

The average result of positive reevaluation was also differentiated by the education of patients (*p* = 0.036) and professional status (*p* = 0.001). Patients with primary/vocational education obtained the highest score in this area (M = 22.01, SD = 2.95), and values for people with secondary and higher education were respectively M = 21.86, SD = 2.91 and M = 20.73, SD = 3.74. Analyzing the professional status, the average score for pensioners (M = 22.26, SD = 2.97) in the scope of positive reevaluation was higher than for working people (M = 20.61; SD = 3.41).

Patients diagnosed with metastasis were characterized by a higher average in the field of helplessness/hopelessness (M = 14.09, SD = 4.58) than patients who were not diagnosed with metastases (M = 12.78, SD = 4.14) (*p* = 0.008).

Chemotherapy influenced the results obtained by patients in the areas of anxiety (*p* = 0.006) and helplessness/hopelessness (p = 0.006). In both cases, higher average values were obtained by people undergoing chemotherapy (M = 17.33, SD = 4.54 and M = 13.92, SD = 4.30) in comparison to people who did not receive chemotherapy (M = 16.02; SD = 4.71 and M = 12.76, SD = 4.52).

Targeted treatment differentiated patients’ results in three areas: fighting spirit (*p* = 0.042), helplessness/hopelessness (*p* = 0.001) and positive reevaluation (*p* = 0.045). Strategies of fighting spirit and positive reevaluation were more often chosen by people not subjected to targeted treatment, for which the values of these areas were respectively: M = 22.39 (SD = 4.04) and M = 21.74 (SD = 3.17) in comparison to the average values of M = 21.03 (SD = 3.61) and M = 20.45 (SD = 3.34) for patients who received targeted treatment. Patients undergoing targeted treatment also have a higher score in the area of helplessness/hopelessness (M = 16.10, SD = 3.13) compared to people who did not receive targeted therapy (M = 13.16, SD = 4.45).

Patients with gastrointestinal cancers obtained the highest values in the MiniMAC test successively in the areas of fighting spirit (M = 21.30, SE = .25), positive reevaluation (M = 21.09, SE = .19), anxiety (M = 17.37, SE = .29) and helplessness and hopelessness (M = 14.22, SE = .27). When analyzing the results of patients due to cancer location, it is noticeable that patients with colorectal cancer obtain the highest values of the MiniMAC test in areas of fighting spirit and positive reevaluation, and the lowest in areas of anxiety and helplessness/hopelessness. Anxiety and helplessness/hopelessness are more characteristic of patients with pancreatic cancer (Table 5).

**Table 5:** Results of the Mini-Mac for patients with gastrointestinal cancers

MiniMAC areas	Total		Pancreas		Stomach		Large intestine		
	M	SE	M	SE	M	SE	M	SE	η^2^
Anxiety	17.37	.29	18.28	.67	17.88	.47	15.95	.29	0.047
Fighting spirit	21.30	.25	19.80	.57	20.69	.40	23.40	.25	0.119
Helplessness/hopelessness	14.22	.27	15.58	.63	14.60	.44	12.46	.28	0.073
Positive reevaluation	21.09	.19	20.20	.45	20.85	.32	22.22	.20	0.058

*M* Estimated marginal mean, *SE* Standard error, η^2^ Absolute impact measure value

## Discussion

At each stage of cancer, patients experience different emotions, which may depend on both the type of cancer, its advancement, the prognosis associated with the treatment, as well as personality factors of the patient. Negative emotions, including anxiety and depression are typical for cancer patients [11]. Cancer can thus deteriorate the quality of life of patients in all areas. Although some studies indicate that a decrease in the quality of life is noticed in patients treated with chemotherapy, studies comparing the quality of life of patients with cancer treated with chemotherapy and patients not treated with chemotherapy do not indicate significant differences among the two groups, which may suggest that the deterioration of the quality of life can be caused by the very fact of making a diagnosis, which causes fear and a feeling of powerlessness, especially in young people [12–14].

Studies show that experiencing symptoms of depression, anger, and anxiety may be affected by experiencing pain [15, 16]. Pain can be felt at any stage of cancer. It occurs in about 50% of patients, and in the advanced period of the disease - in over 75%. Pain may be caused by the neoplastic process, cancer cachexia, and may also be the result of anticancer treatment [17]. Pain can be felt by patients in a variety of ways, and the patients’ response to the pain sensation often depends on personality factors.

Patients with gastrointestinal cancers assign the greatest role in controlling pain to internal factors, similar to patients with lung cancer [18], patients with breast cancer in the study conducted by Czerw A. et al. [19] and patients with prostate cancer [20]. What is interesting, the main socio-economic variables, differentiating the attribution of the impact of individual areas on the degree of pain control were education and net income per household member. People with higher education and higher income attributed less influence to these areas [21].

Patients with gastrointestinal cancers usually cope with pain by declaring coping and praying/hoping. Patients with lung cancer [18] and patients with breast cancer [19], similarly to patients with gastrointestinal cancer in the author’s study, usually choose the strategy of declaring coping, and the results in both patients with breast and lung cancer are differentiated by education and income [22].

The selection of strategies for coping with pain by patients with gastrointestinal cancers in the author’s study is affected by all socio-economic variables studied (gender, age, education, professional status, income) apart from the place of residence. The results are also differentiated by the diagnosis of metastases, chemotherapy, radiotherapeutic and targeted therapy. A significant difference was observed between the patients analyzed due to the location of cancer in the area of catastrophizing. The author’s own study indicates that the catastrophizing strategy is most often chosen by patients with pancreatic cancer, and much less frequently by patients with colorectal cancer.

The catastrophizing strategy significantly reduces the quality of life assessed by patients. Studies indicate that patients who require surgery are often using the catastrophizing strategy [23]. A strong relationship of anxiety with the use of the catastrophizing strategy is also emphasized, which also translates into a reduction in the sense of self-efficacy as to the impact on health or not following pharmacological recommendations [24–26]. Among patients with gastrointestinal cancers in the author’s study, the catastrophizing strategy was selected more often by younger patients, patients with lower income, with metastases, as well as patients treated with chemotherapy and receiving targeted therapy.

The quality of life with the illness (in this case cancer) assessed by patients is strongly influenced by the acceptance of this illness. Chronic diseases, associated with numerous limitations of many activities, may determine the difficulty in accepting the illness [27]. B.J. Felton et al. indicate that low acceptance of the illness is strongly associated with negative attitudes towards the perceived illness, which in turn affects the deepening of symptoms and faster progression of the illness [28]. In addition to the acceptance of the illness and the strength of the symptoms, the impact of the level of acceptance of the illness on the daily activity of patients can be noticed [29, 30]. The more the patient accepts their illness, the more often he or she takes active actions, aimed at minimizing the feeling of pain or changing his or her behavior for those that will help maintain a relatively good health [31, 32]. However, studies suggests that some patients who accept their health status may not undertake pro-health activities. These patients try to accept pain and limitations resulting from the illness, and agree with the fact that their health will deteriorate [33].

Among patients suffering from gastrointestinal cancer, the average value of illness acceptance was the lowest in the group of patients with pancreatic cancer, and the highest in the group of people with colorectal cancer. The study of patients with various types of cancer indicates that the highest level of acceptance of the illness, i.e. the best ability to adapt to cancer, is characteristic for women with cancer of reproductive organs in comparison to patients with gastrointestinal cancers, including colorectal cancer, stomach cancer and pancreatic cancer [34]. In turn, B.J. Felton et al., examining chronically ill persons, indicated that the average value of the AIS test for these patients was M = 28.08 [35].

Among patients with various cancers (breast, lung, prostate, large intestine) the main socioeconomic factor differentiating the results obtained in the AIS test was income. In all groups, there was a linear relationship between the increase in net income per household member and the result of the AIS test [36], and this correlation was also obtained in the author’s study. In addition, among patients with gastrointestinal cancers in the author’s study, the relationship between the level of acceptance of the illness with gender, professional status and the diagnosis of metastasis was noted. Men, working persons and patients who were not diagnosed with metastases obtained a higher average result in the AIS test.

Analyzing adaptation to the illness, patients with gastrointestinal cancers obtained the highest values in the MiniMAC test in the area of fighting spirit, and the lowest in the area of helplessness/hopelessness. The average value of the area of fighting spirit was the highest in patients with colorectal cancer. Anxiety and helplessness/hopelessness are characteristic for patients with pancreatic cancer. In the study comparing various neoplastic changes, in the areas of anxiety and hopelessness/hopelessness, the highest scores were obtained successively by the respondents with lung cancer, colorectal cancer, breast cancer and prostate cancer. In the area of fighting spirit there was no reversal of results. Higher results in this area were obtained by respondents who had breast cancer and patients with colorectal cancer, and lower - by responders with prostate cancer and lung cancer [37].

Among the variables studied, mental adaptation to cancer in patients with gastrointestinal cancers is influenced by patients’ gender, age, education, professional status, diagnosis of metastasis, chemotherapeutic treatment and targeted treatment.

A study of 572 patients of the Oncology Center in Warsaw diagnosed with cancer of the reproductive system, cancer of the head and neck, gastrointestinal cancer, breast cancer, urological cancer, cancer of the tissues and nervous system, lung cancer indicated a strong relationship of negative emotions felt in connection with the disease with gender. Women are more often characterized by catastrophizing, higher levels of depression and anxiety compared to men, they show greater intensification of anxiety, helplessness/hopelessness, but also fighting spirit and positive reevaluation [11, 38, 39].

A study of 220 patients with various cancers (stomach cancer, genital cancer, pancreatic cancer, colorectal cancer, prostate cancer) covered by palliative care indicates that mental adaptation to cancer is strongly influenced by the degree of its acceptance. With the increase in acceptance of the illness, the intensity of fighting spirit decreases and the sense of helplessness/hopelessness intensifies [34].

Intensification of the strategy of helplessness/hopelessness is caused by feelings of anxiety and symptoms of depression. The smaller the feeling of anxiety and negative emotions associated with the illness, the more often patients adopt the strategy of fighting spirit. Attitudes towards the illness constitute an expression of how patients cope with the negative effects of cancer: pain, malaise, changes in life [11]. The use of active strategies, such as fighting spirit or positive reevaluation, affects the higher quality of life experienced by patients [6, 40].

## Conclusions

1. Patients with gastrointestinal cancers control pain mainly by internal factors, however, the areas of influence of doctors and random events were most often differentiated by socio-economic variables.
2. The strategy of coping with pain most often selected by patients with gastrointestinal cancers is declaring coping, and the choice of strategy was influenced by all socio-economic variables apart from the place of residence, as well as diagnosis of metastasis and the type of therapy used.
3. Among patients with gastrointestinal cancers, patients with colorectal cancer have the highest level of acceptance of illness, while patients with pancreatic cancer – the lowest.
4. In the area of mental adaptation to illness, patients most often declared fighting spirit, however patients with pancreatic cancer were characterized by significant anxiety and helplessness/hopelessness.

## Data Availability

The data analyzed during the current study are available from the corresponding author.

## Abbreviations

BPCQ: The Beliefs about Pain Control Questionnaire
CSQ: The Pain Coping Strategies Questionnaire
AIS: Approval Illness Scale
MiniMAC: Mental Adjustment to Cancer
M: Mean
SD: Standard deviation
p: Probability value
